# Supplementary material for: Direct genome-scale screening of Gluconobacter oxydans B58 for rare earth element bioleaching
Source: Commun Biol. 2025 Apr 30;8:682. doi: 10.1038/s42003-025-08061-4 (PMC12041372; doi:10.1038/s42003-025-08061-4)
Supplement: Supplementary file 1 — Supplementary Information [file 42003_2025_8061_MOESM1_ESM.pdf]

Supplementary Information for:

Direct Genome-Scale Screening of *Gluconobacter oxydans* B58 for Rare Earth Element Bioleaching

Sabrina Marecos[1]; Brooke Pian[1*]; Sean A. Medin[1*]; Alexa Schmitz[1*]; Melinna Andrade[1]; Mingming Wu[1]; J. Brian Balta[2]; Esteban Gazel[2]; Megan Holycross[2]; Matthew C. Reid[3]; Buz Barstow[1,‡]

[1]Department of Biological and Environmental Engineering, Cornell University, Ithaca, NY 14853, USA

[2]Department of Earth and Atmospheric Sciences, Cornell University, Ithaca, NY 14853, USA

[3]School of Civil and Environmental Engineering, Cornell University, Ithaca, NY 14853, USA

[‡]Corresponding author: Buz Barstow, 228 Riley-Robb Hall, Cornell University, Ithaca, NY 14853, USA; bmb35@cornell.edu

*Present address: REEgen, Inc., Praxis Center for Venture Development, Cornell University, Ithaca, NY 14853, USA

Supplementary Information Figures

Figure S1. Comparison of bioleaching by identified gene disruption mutants with *G. oxydans* wild-type.

Figure S2. Validation of *G. oxydans* engineered strains.

Supplementary Information Tables

Table S1. Changes produced by gene disruptions selected for direct measurement of bioleaching after cell density normalization.

Table S2. Strains and plasmids used in this study.

Table S3. Oligonucleotides used in this study.

Supplementary Information Datasets

Supplementary Data 1. Catalog of *G. oxydans* B58 Quality-Controlled Whole Genome Knockout Collection.

Supplementary Data 2. Characterization of Synthetic Monazite Powders.

Supplementary Data 3. *G. oxydans* B58 Quality-Controlled Whole Genome Knockout Collection Screening Data and Analysis.

Supplementary Data 4. Direct Measurement of Bioleaching Data.

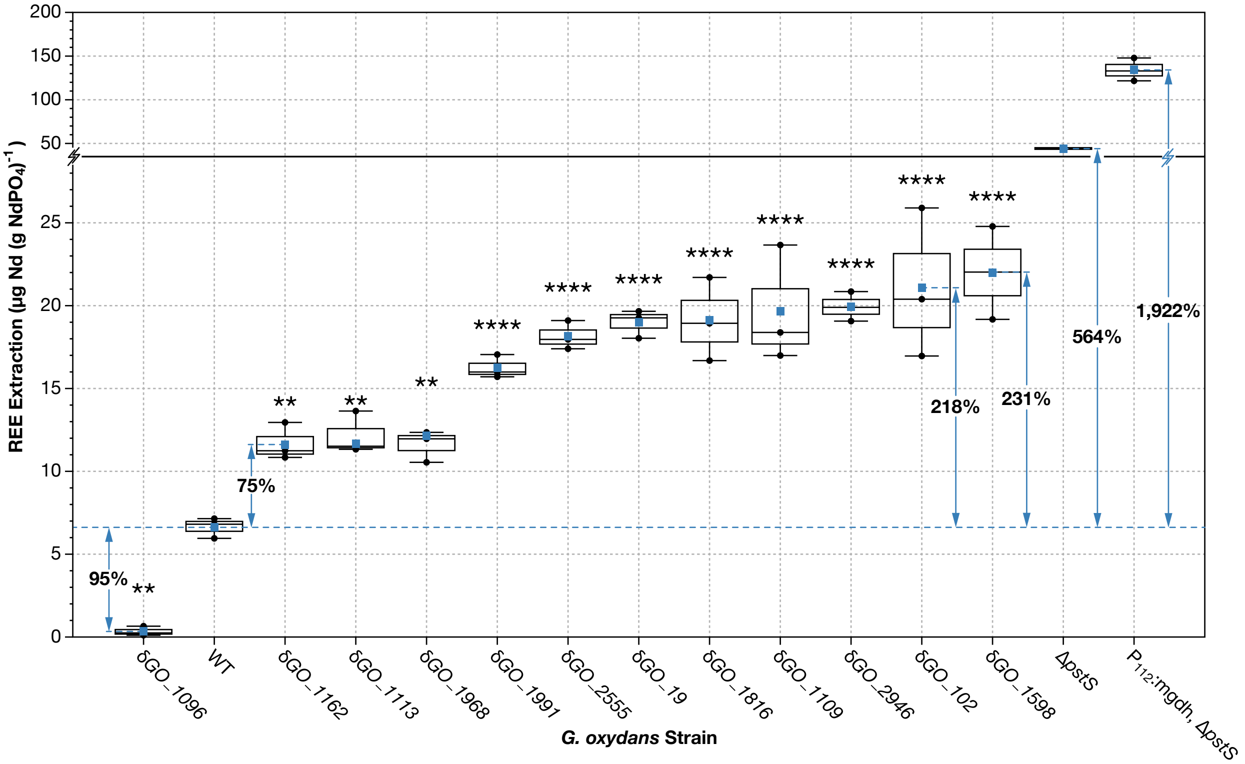

Figure S1. Comparison of bioleaching by identified gene disruption mutants with *G. oxydans* wild-type. Direct measurement of bioleaching extraction was performed on disruption mutants of interest after bioleaching experiments with synthetic monazite using ICP-MS analysis. REE extractions were analyzed with pairwise comparisons among disruption mutants (n = 3) and the wild-type strain (n = 3). Extraction levels significantly different from *G. oxydans* wild-type are labeled with asterisks (* $p < 0.05$; ** $p < 0.01$; *** $p < 0.001$; **** $p < 0.0001$) and represent statistical significance after Bonferroni correction ($N = 12$). Blue squares indicate the mean extraction for each mutant, the center line denotes the median, boxes show the upper and lower quartiles, and whiskers extend to the range of data points within 1.5 times the interquartile range. All strains analyzed were statistically significant. Disruption mutant δ*GO_1096* demonstrated a 95% reduction in extraction, while all other disruption mutants showed increased extraction.

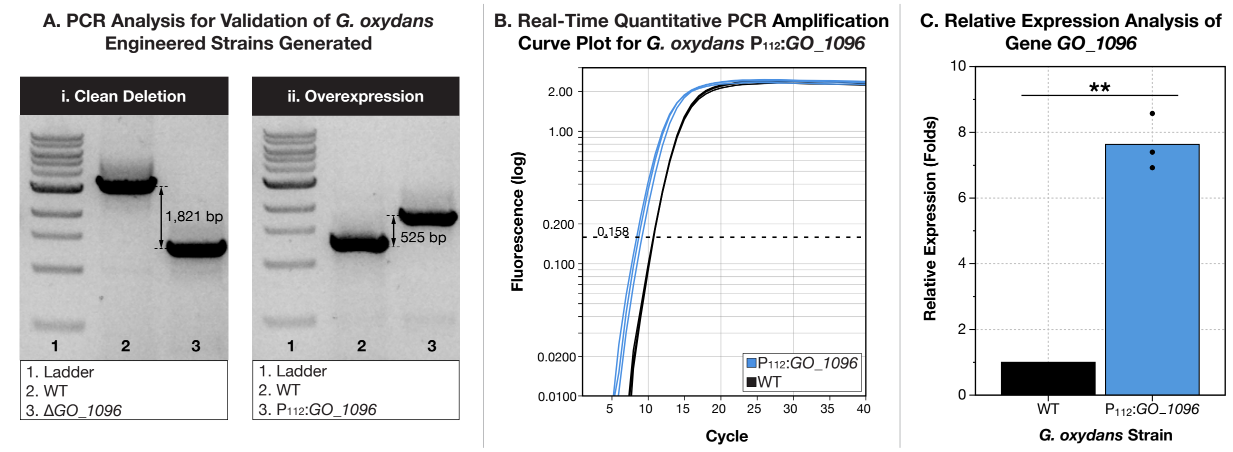

Figure S2. Validation of *G. oxydans* engineered strains. (**A**) PCR confirmation of engineered strains, showing successful clean deletion (left) and overexpression (right) of *GO_1096*. (**B**) Real-Time quantitative PCR (RT-qPCR) amplification curves verifying increased expression of *GO_1096* in the overexpression strain (P_{112}:*GO_1096*) compared to the wild-type. (**C**) Relative expression analysis demonstrating significant upregulation of *GO_1096* in P_{112}:*GO_1096*, confirming successful transcriptional enhancement. Statistical significance determined through unpaired *t*-test comparing to WT is labeled with asterisks (** $p < 0.01$).

GO Strain	Extraction Against WT	SD	Significance
δGO_1096	-95	0.16	***
δGO_1162	+10	1.00	ns
δGO_1991	+10	0.91	ns
δGO_1968	+21	1.14	ns
δGO_2555	+25	0.62	*
δGO_1113	+27	0.23	*
δGO_1816	+34	1.01	*
δGO_1598	+36	1.05	*
δGO_2946	+41	0.72	*
δGO_19	+49	0.33	**
δGO_102	+50	2.03	ns
δGO_1109	+50	1.82	*

Table S1. Changes produced by gene disruptions selected for direct measurement of bioleaching after cell density normalization. This table presents the percentage change in extraction efficiency produced by each gene disruption compared to the wild-type strain (WT) after normalization of the cell density, where positive values indicate enhanced bioleaching, and negative values denote a reduction. Additionally, the standard deviation (SD) is listed, along with statistical significance determined through unpaired *t*-tests performed against WT. Significance levels are indicated by asterisks (*$p < 0.05$; **$p < 0.01$; ***$p < 0.001$, ****$p < 0.0001$) or "ns" if $p > 0.05$).

Strain or Plasmid	Characteristics	Source
G. oxydans B58	Wild-type (ATCC 9844)	Laboratory stock
pKOS6b	*E. coli* vector, *codBA* integrated, Km^R, confers 5-fluorocytosine (FC) sensitivity	Laboratory stock
pKOS6b-KO_GO1096	pKOS6b vector for deletion of *GO_1096* in *G. oxydans* B58, Km^R, FC^S	This study
pKOS6b-P_{112}_GO1096	pKOS6b vector for insertion of P_{112} promoter in *G. oxydans* B58, Km^R, FC^S	This study
Δ*GO_1096*	B58 derivative with deletion of *GO_1096*	This study
P_{112}:*GO_1096*	B58 derivative with up-regulation of *GO_1096* via P_{112} promoter	This study

Table S2. Strains and plasmids used in this study.

Description	Forward Primer	Reverse Primer
**Deletion of *GO_1096***		
Amplification of upstream homology flank	tgacatgattacgaattcgagctcggtacccggggatcctctagatgccgtgcgtggcg	ccagtcctgaaaaggacggagtggtcttccagacgggagg
Amplification of downstream homology flank	cctcccgtctggaagaccactccgtccttttcaggactggt	acgacggccagtgccaagcttgcatgcctgcaggtcgactctagagctgcagtgccggc
Genotype Validation	ccctgatggggagaggggac	cagcaacatgccagtgacg
**Up-regulation of *GO_1096* via P_{112} promoter**		
Amplification of upstream homology flank	tgacatgattacgaattcgagctcggtacccggggatcctctagatgccgtgcgtggcg	accgttccctgctggaactggtggtcttccagacgggagg
Amplification of P_{112} promoter	cctcccgtctggaagaccaccagttccagcagggaacgg	gcgatattacggagttccatggaactgactcctgatttcgttct
Amplification of downstream homology flank	cgaaatcaggagtcagttccatggaactccgtaatatcgccatca	acgacggccagtgccaagcttgcatgcctgcaggtcgactctagagacggtcacggatctggc
Genotype Validation	ccctgatggggagaggggac	gacggtcacggatctggc
cDNA amplification for gene expression analysis	aacttcagcttggcgttctc	acgtcaaccaggacttcttc

Table S3. Oligonucleotides used in this study.
