# Supplementary material for: Direct genome-scale screening of Gluconobacter oxydans B58 for rare earth element bioleaching
Source: Commun Biol. 2025 Apr 30;8:682. doi: 10.1038/s42003-025-08061-4 (PMC12041372; doi:10.1038/s42003-025-08061-4)
Supplement: Supplementary file 2 — Description of Additional Supplementary Files [file 42003_2025_8061_MOESM2_ESM.docx]

Description of Additional Supplementary Files

**File name:** Supplementary Data 1

**Description:** Catalog of G. *oxydans* B58 QualityControlled Whole-Genome Knockout Collection.

**File name:** Supplementary Data 2

**Description:** Characterization of Synthetic Monazite Powders.

**File name:** Supplementary Data 3

**Description:** G. *oxydans* B58 Quality-Controlled Whole-Genome Knockout Collection Screening Data and Analysis.

**File name:** Supplementary Data 4

**Description:** Direct Measurement of Bioleaching Data.
